# Betaine-Based Deep Eutectic Solvent as a New Media for Laccase-Catalyzed Template-Guided Polymerization/Copolymerization of Aniline and 3-Aminobenzoic Acid

**DOI:** 10.3390/ijms231911409

**Published:** 2022-09-27

**Authors:** Irina Vasil’eva, Olga Morozova, Galina Shumakovich, Alexander Yaropolov

**Affiliations:** A. N. Bach Institute of Biochemistry, Research Center of Biotechnology of the Russian Academy of Sciences, 119071 Moscow, Russia

**Keywords:** deep eutectic solvent, template-guided enzymatic polymerization, laccase, aniline, 3-amonobenzoic acid, copolymer, antimicrobial properties

## Abstract

Deep eutectic solvents (DESs) can compensate for some of the major drawbacks of traditional organic solvents and ionic liquids and meet all requirements of green chemistry. However, the potential of their use as a medium for biocatalytic reactions has not been adequately studied. In this work we used the DES betaine-glycerol with a molar ratio of 1:2 as co-solvent for enzymatic template-guided polymerization/copolymerization of aniline (ANI) and 3-aminobenzoic acid (3ABA). The laccase from the basidial fungus *Trametes hirsuta* and air oxygen served as catalyst and oxidant, respectively. Sodium polystyrene sulfonate (PSS) was used as template. Interpolyelectrolyte complexes of homopolymers polyaniline (PANI) and poly(3-aminobenzoic acid) (P3ABA) and copolymer poly(aniline-co-3-aminobenzoic acid) (P(ANI-3ABA)) were prepared and their physico-chemical properties were studied by UV-Vis and FTIR spectroscopy and cyclic voltammetry. According to the results obtained by atomic force microscopy, PANI/PSS had a granular shape, P(ANI-3ABA)/PSS had a spherical shape and P3ABA/PSS had a spindle-like shape. The copolymer showed a greater antimicrobial activity against *Escherichia coli* and *Staphylcocus aureus* as compared with the homopolymers. The minimal inhibitory concentration of the P(ANI-3ABA)/PSS against the gram-positive bacterium *S. aureus* was 0.125 mg mL^−1^.

## 1. Introduction

The term “green chemistry” implies the development of a strategy aimed at designing new products that minimize or eliminate the use and generation of hazardous substances. In the last few years, deep eutectic solvents (DESs) have drawn a great attention as an alternative to traditional organic solvents and ionic liquids. The term ‘deep eutectic solvents’ was first coined by Abbott et al. [1] in 2003, however, there is still no clear definition [2]. DESs are formed by mixing particular ratios of two or more substances. The resultant liquid has a lower melting temperature as compared to those of the DES components [1]. As a rule, the DES components—hydrogen bond acceptor (HBA) and hydrogen bond donor (HBD)—are natural biodegradable organic substances [3,4,5]. The properties of DESs depend on the nature of HBA and HBD, their molar ratio and water content [6]. The number of HBA and HBD combinations is extremely large, which enables the formation of DESs with different properties (hydrophilic, hydrophobic, acidic, alkaline, neutral, etc.) and tailoring DESs for specific applications. DESs are similar to ionic liquids. However, they have some advantages, like ease of preparation, low preparation costs, low toxicity, biodegradability and the possibility of varying characteristics with changes of DES components and their ratio [4]. The potential of DESs results from unique properties of these solvents like nonflammability, low vapor pressure, thermal and chemical stability, a wide polarity range and high solubility of numerous compounds in them, as well as from their high extraction and stabilizing abilities [4,7,8]. Thus, DESs are promising green solvents which can find applications in many fields, including the pharmaceutical, food and cosmetics industries [9,10,11]. They can also be used for extractions and separations [10,12,13,14,15,16], biomass processing [11,17,18], dissolution of metals [11,19], in material and polymer chemistry [20,21,22,23,24] and as a reaction medium for organic reactions [11,25,26,27,28]. DESs have proved to be a good alternative to traditional organic solvents and ionic liquids in many biocatalytic processes [29]. The use of DESs as a medium for biocatalytic reactions can be promising for the biotransformation of slightly soluble substances and the design of products with novel properties.

The interest in the use of enzymes in organic synthesis has been growing in recent years [30,31,32]. In terms of the environment, enzymes are natural and renewable and, hence, belong to green catalysts [30]. In addition, enzymatic reactions proceed under ‘mild’ operating conditions, namely at pH values close to neutral, room temperature, under atmospheric pressure and in the absence of toxic organic solvents and meet the requirements of sustainable chemistry [29,33,34]. The use of DESs and water (buffer)/DES mixtures as media for enzymatic reactions is currently reported for a limited range of biocatalysts [29,33,34,35,36,37].

Of special interest as a biocatalyst for fine organic synthesis is laccase [38,39,40,41]. Laccase (*p*-diphenol:oxygen oxidoreductase, EC 1.10.3.2), which belongs to the blue multicopper oxidase family, catalyzes oxidation of a wide range of organic compounds with atmospheric oxygen and its reduction to water [42,43]. Laccase-catalyzed oxidation of aromatic compounds proceeds via a radical mechanism with the subsequent combination of intermediates, which results in the formation of oligomeric and polymeric products [38,44,45]. Laccase-catalyzed reactions are usually carried out in buffer solutions or, if substrates are poorly dissolved in water, in organic-water solutions.

Aniline (ANI) is a promising substrate for laccase, as its oxidation results in the formation of polyaniline (PANI), which is an important conducting polymer [46]. PANI has a great potential for technological applications due to the ease of production, low cost of the monomer, environmental stability and ability to change electrical and optical characteristics when varying the degree of protonation and oxidation [46,47,48,49,50,51]. In addition, PANI and its functionalized derivatives show antimicrobial properties [52,53,54,55,56,57], which enables their use for making film coats to protect various surfaces against bacterial contamination [58].

The properties of PANI can be varied in a wide range depending on synthesis conditions [59,60]. PANI is generally produced by electrochemical or chemical methods [46,61,62], but both methods have some drawbacks. The electrochemical method is not always suitable as monomer polymerization proceeds on electroconducting substrates of limited sizes, and the chemical synthesis requires great amounts of oxidants, whose reduction products should be disposed of. In terms of the environment, the enzymatic synthesis of PANI is a good alternative to traditional methods [63,64,65,66,67,68].

As mentioned above, the use of DES and DES/buffer mixtures in enzymatic reactions is reported for a limited range of biocatalysts. Although eutectic media have same advantages, e.g., a higher solubility of substrates and/or products, their wide application is limited by a lower stability of biocatalysts.

In this work we have investigated the influence of the DES betaine-glycerol (molar ratio 1:2) on the catalytic activity and stability of the laccase from the fungus *Trametes hirsuta*, performed an enzymatic template-guided polymerization/copolymerization of aniline and 3-aminobenzoic acid in a DES/buffer mixture (60/40 vol.%) and characterized the resulting products.

## 2. Results and Discussion

**Figure 1:**
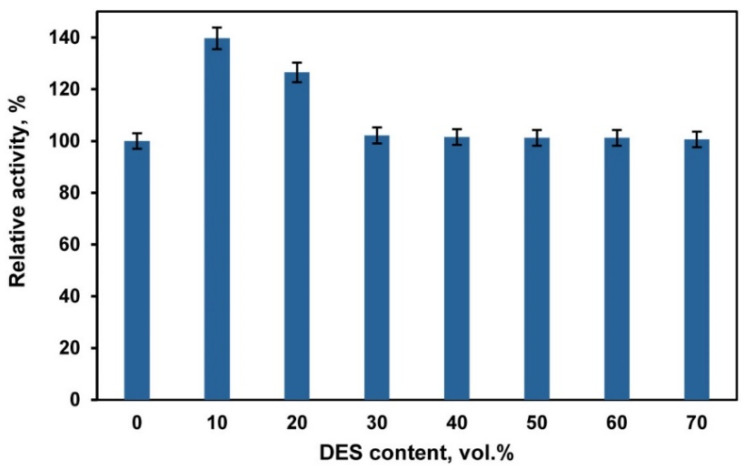
Laccase activity in betaine-glycerol (1:2)/buffer mixtures with different volume ratios of the components at room temperature (21–22 °C). Laccase activity in the buffer was considered to be 100%.

**Figure 2:**
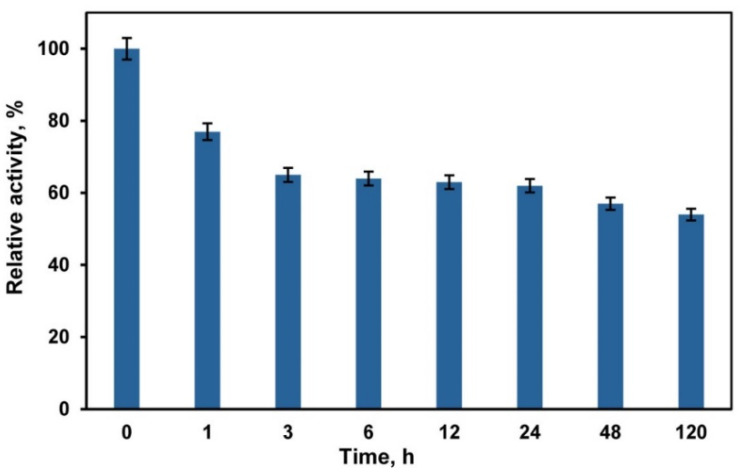
Laccase stability in betaine-glycerol (1:2)/buffer (60/40 vol.%) mixture. Laccase activity in the DES/buffer mixture at the initial time was considered to be 100%.

The use of deep eutectic solvents (DESs) and DES/buffer mixtures as a medium for biocatalytic reactions requires taking into account their effect on the activity and stability of enzymes. Choline chloride-based DESs are in most common use [29,34,35,36,37]. However, chloride ions inhibit the activity of fungal laccases [69]. Therefore, in our experiments we used a DES composed of betaine (HBA) and glycerol (HBD) with a molar ratio of 1:2. Betaine-glycerol (1:2) composition like most DESs is a viscous liquid. The viscosity can be markedly decreased by adding water [70]. Our studies of laccase activity in DES/buffer mixtures with a varied content of DES (Figure 1) showed that the enzyme activity increased at a DES content of 10–20 vol.%. A similar activation of laccases at a rather low DES content (up to 20 vol.%) in the reaction medium is reported in [71,72,73]. However, Hammond et al. [74] showed that DES properties were retained in the DES/H_2_O mixture if the water content is less than 42 wt.%, and, at higher contents of water, the DES/H_2_O mixture should be considered as a water solution of DES components. Therefore, we chose a DES/buffer mixture with a volume ratio of 60/40 vol.% to perform laccase-catalyzed polymerization/copolymerization of aniline (ANI) and 3-aminobenzoic acid (3ABA). At this DES/buffer ratio the mass content of water was ~35 wt.%, which enabled us to assume that the structure of DES remained intact. Besides this DES/buffer mixture was less viscous as compared to pure DES. Studies of laccase stability showed (Figure 2) that the enzyme retains ~60% of its activity after 24 h incubation in DES/buffer (60/40 vol.%) mixture.

**Figure 3:**
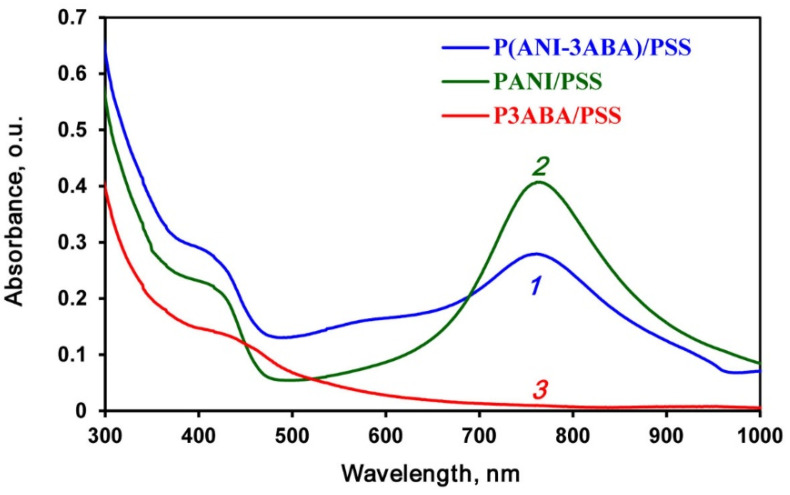
UV–visible spectra of the P(ANI-3ABA)/PSS copolymer (*1*), and PANI/PSS (*2*) and P3ABA/PSS (*3*) homopolymers. The samples were diluted with buffer (1:10).

Using enzymatic polymerization/copolymerization of ANI and 3ABA on a PSS template (Supplementary Figure S1) in DES/buffer (60/40 vol.%) mixture (pH 3.5), we obtained PANI/PSS, P3ABA/PSS and P(ANI-3ABA)/PSS interpolyelectrolite complexes, whose UV–visible spectra are shown in Figure 3. It is noteworthy that the PANI/PSS homopolymer and copolymer had green color typical of conducting polyanilines, while the P3ABA/PSS homopolymer was of light brown color.

The PANI/PSS spectrum (Figure 3, line *2*) has two wide absorbance bands with maxima at 762 and 405 nm, which indicates the formation of a polaron in emeraldine salt of PANI. It should be mentioned that the spectrum is no different from the spectrum of PANI/PSS synthesized in the aqua medium using laccase [75]. The absorbance band attributed to the polaron in the spectrum of P(ANI-3ABA)/PSS are less distinct, which is due to the side electron acceptor (carboxyl) group of aminobenzoic acid in the main chain of the copolymer. The spectrum of P3ABA/PSS has no bands assigned to the polaron, which seems to be related to the fact that the charge carriers are more localized on the nitrogen atoms. This effect can be explained by the formation of a five-member ring caused by electrostatic interaction between COO^−^ groups and the cationic radical nitrogen atoms [76]. Very low conductivity of P3ABA/PSS (~ 10^−10^ S cm^−1^) seems to be due to this effect. At the same time, the conductivity of PANI/PSS and P(ANI-3ABA)/PSS was much higher (2.2 × 10^−3^ and 1.3 × 10^−5^ S cm^−1^, respectively).

**Figure 4:**
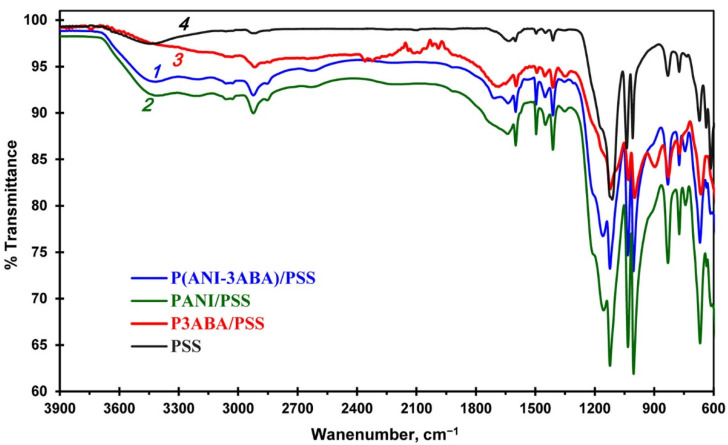
ATR-FTIR spectra of the P(ANI-3ABA)/PSS copolymer (*1*), PANI/PSS (*2*) and P3ABA/PSS (*3*) homopolymers, and PSS template (*4*).

The molecular structures of the interpolyelectrolite complexes PANI/PSS, P3ABA/PSS and P(ANI-3ABA)/PSS were studied using FTIR-spectroscopy, which enabled us to get additional information about the polymers synthesized in the DES/buffer mixture (Figure 4). The bands at 970–1200 cm^−1^ in the spectra of all the polymers are assigned to the S=O bonds stretching in the PSS template, which indicates the formation of durable polymer/PSS complexes. The characteristic peaks at 1500 and 1600 cm^−1^ are assigned to the C–C aromatic ring stretching of the benzenoid diamine units and to the stretching of both C=N and C=C of the quinoid diimine units, respectively. In addition, an additional band is observed in the spectra of P(ANI-3ABA)/PSS (1705 cm^−1^) and homopolymer P3ABA/PSS (1690 cm^−1^). This can be explained by C=O stretching in the carboxyl groups which is absent in the PANI/PSS spectrum. The vibrations in the range of 650–850 cm^−1^ are attributed to the C–H in-plane bending vibration of the benzene rings. The homopolymer P3ABA/PSS spectrum has a band at 900 cm^−1^, which corresponds to the C–H out-of-plane bending vibration of the trisubstituted benzene ring.

**Figure 5:**
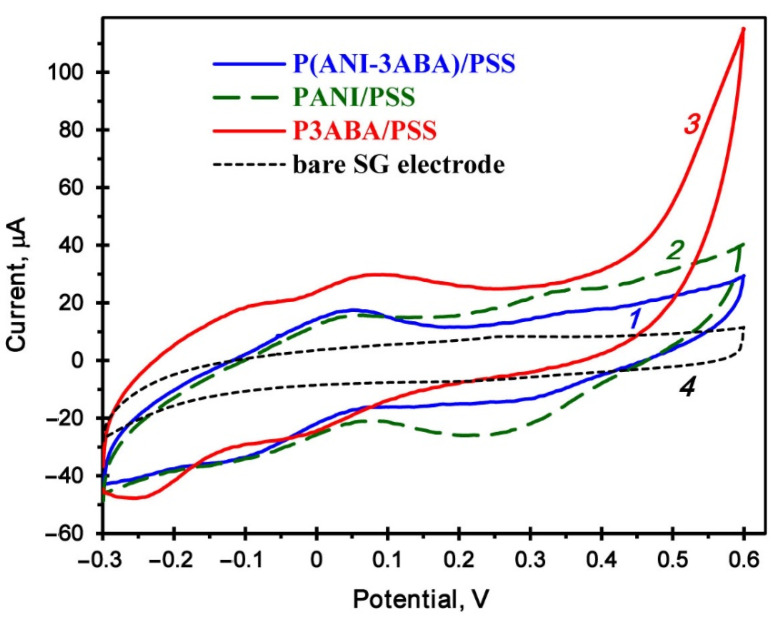
Cyclic voltammograms of the copolymer P(ANI-3ABA)/PSS (*1*), homopolymers PANI/PSS (*2*) and P3ABA/PSS (*3*), and the bare SG electrode (*4*) recorded in 0.1 M Na-citrate-phosphate buffer, pH 3.5 at a scan rate of 50 mV s^−1^.

The homopolymers and copolymer were tested by cyclic voltammetry (CV) on a spectroscopic graphite (SG) electrode. The studies showed that they are all electrochemically active (Figure 5). Redox conversions of the benzenoid and quinoid units are marked with couples of pseudoreversible peaks, whose characteristics are given in Table 1, where ∆E is the difference in potentials for the maximum of anodic (E_a_) and cathodic (E_c_) peaks, and E_mp_ is the middle point potential. No redox conversions were registered on the bare SG electrode.

**Figure 6:**
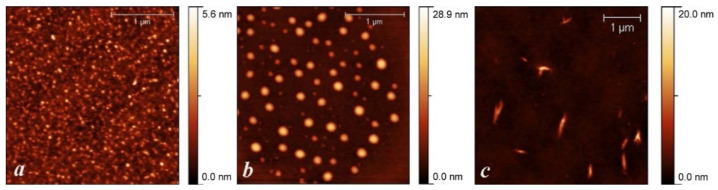
AFM-images PANI/PSS (**a**), P(ANI-3ABA)/PSS (**b**), P3ABA/PSS (**c**).

Studies of the films of the polymer/PSS complexes formed on the surface of highly oriented pyrolytic graphite (Figure 6) showed significant morphological differences. PANI/PSS has a morphology with small, aggregated granules with a diameter of 20–50 nm. P(ANI-3ABA)/PSS nanoparticles have a spherical shape with a diameter varying from 60 to 180 nm, while P3ABA/PSS particles have a spindle-like shape with a diameter of 60–90 nm and length of 300–500 nm.

The synthesized polymers were tested against microbial activities of the bacteria *E. coli* and *S. aureus* and the yeast *Yarrowia lipolytica*. The PSS template had no effect on the growth of the microbes. The PANI/PSS homopolymer at a maximum concentration obtained in the study (2.9 mg mL^−1^) significantly but not completely suppressed the growth of *E. coli* and *S. aureus*. The minimal inhibitory concentration (MIC) of the P(ANI-3ABA)/PSS copolymer against the gram-positive bacterium *S. aureus* was 0.125 mg mL^−1^. In addition, the copolymer essentially suppressed growth of the gram-negative bacterium *E. coli* at a concentration of 0.5 mg mL^−1^. MIC of the P3ABA/PSS homopolymer against both *S. aureus* and *E. coli* was 2.93 mg mL^−1^. In addition, at a concentration of 5.85 mg mL^−1^ P3ABA/PSS suppressed growth of the yeast *Y. lipolytica*, whereas PANI/PSS and P(ANI-3ABA)/PSS showed no effect on the yeast growth.

It is noteworthy that MIC values for polyaniline, poly(3-aminobenzoic acid) and poly(aniline-co-3-aminobenzoic acid) synthesized by the traditional chemical method [54] were 10, 2.5 and 1.25 mg mL^−1^ against *E. coli* and >10, 2.5 and 1.2 mg mL^−1^ against *S. aureus*, respectively, which makes it clear that polymer/PSS complexes enzymatically synthesized in the DES/buffer mixture are more efficient as growth inhibitors of gram-positive and gram-negative bacteria.

Conducting polymers, in particular polyaniline and its derivatives, are rather new antibacterial agents, whose mechanism of action has not been adequately studied yet, but several possible antibacterial mechanisms have been proposed. Sheshardi & Bhat [52], who were the first to show the antibacterial action of chemically synthesized PANI against the bacteria *S. aureus* and *E. coli*, and the fungus *Candida albicans*, attributed it to the destruction of cell walls caused by electrostatic interaction between the polymer and bacteria. Recently, other mechanisms were reported in [77], according to which the antimicrobial action of PANI is related to the production of hydrogen peroxide that causes the formation of hydroxyl radicals, while P3ABA disrupts metabolic and respiratory machinery by targeting ATP synthase and causes acid stress. Higher antimicrobial activity of P(ANI-3ABA)/PSS as compared to homopolymers can probably be explained by involving all the three mechanisms.

## 3. Materials and Methods

### 3.1. Materials

KH_2_PO_4_, NaOH, citric acid, 2,2-azino-bis(3-ethyl-benzthiazoline-6-sulfonate (ABTS), betaine, aniline (ANI), poly(sodium 4-styrenesulfonate) solution (PSS, 30 wt.% in H_2_O) (Sigma-Aldrich, Saint Louis, MO, USA), glycerol (99%) (Panreac Quimica SA, Barselona, Spain), 3-aminobenzoic acid (3ABA) (Acros organics, Geel, Belgium) were used without further purification. Aniline (ANI) was distilled in vacuo before use. All the solutions were prepared using water purified with a Simplicity® Water Purification System (Merck KGaA, Darmstadt, Germany).

### 3.2. Enzyme

A laccase from the fungus *Trametes hirsuta* (Wulfen) Pilát CF-28 was purified to homogeneity as described previously [78]. The laccase activity was measured spectrophotometrically using 1 mM ABTS as chromogenic substrate (λ = 420 nm, ε = 36,000 M^−1^cm^−1^) at 24 °C in 0.1 M Na-citrate-phosphate buffer, pH 4.5. One unit of activity is defined as the amount of laccase oxidizing 1 µM of substrate per min. The specific activity of the enzyme stock solution was about 145 U mg^−1^ of protein. Protein concentration was determined as described in [79] by the difference in the optical density of the protein solution at 228.5 and 234.5 nm using a bovine serum albumin (BSA) as standard. The protein concentration was ca. 7.8 mg mL^−1^.

### 3.3. Preparation of DES and DES/Buffer Mixture

DES betaine/glycerol (molar ratio 1:2) was obtained using a thermal mixing procedure at 40 °C with 300 rpm agitation. DES/buffer binary mixture was obtained by adding 4 mL of 0.1 M Na-citrate-phosphate buffer (pH 3.5) to 6 mL of DES.

### 3.4. Enzymatic Polymerization/Copolymerization in DES/Buffer Mixture

The enzymatic copolymerization of ANI and 3ABA (molar ratio 1:1) in DES/buffer mixture on PSS template was carried out as follows: 0.152 g of a 30% aqueous solution of PSS (concentration 20 mM based on the monomeric repeat unit) was added to 10 mL of DES/buffer mixture (60/40 vol.%) and stirred for 30 min. The 10 µL of ANI and 0.016 g of 3ABA were added and the resultant solution was stirred for another 30 min and its pH was adjusted to 3.5 with phosphoric acid. Polymerization was initiated by adding a laccase stock solution. The specific activity of the enzyme in the reaction mixture was ~1.0 U mL^−1^. The reaction was performed at room temperature (21–22 °C) under aerobic conditions and with constant stirring on a magnetic stirrer for 24 h. The resulting P(ANI-3ABA)/PSS copolymer was purified by dialysis against acidified deionized water (pH 3.5) in order to remove excess monomers and DES components.

The synthesis of PANI/PSS and P3ABA/PSS homopolymers was performed under the same conditions with the appropriate monomers (20 µL ANI or 0.032 g 3ABA).

### 3.5. Characterization of Products

The absorption spectra were recorded with a Shimadzu UV 1240 mini spectrophotometer (Japan) in a quartz cuvette with an optical path length of 1 cm. ATR FTIR-spectra were recorded on a Spectrum Two™ FT-IR spectrometer (PerkinElmer Inc., Waltham, MA, USA).

The morphology of the products was studied using a SmartSPM 1000 Scanning Probe Microscope (AIST-NT, Moscow, Russia) on the surface of highly oriented pyrolytic graphite (Advanced Technologies Centre, Russia). For AFM studies, samples were diluted 10 times with deionized water.

Four-point conductivity measurements were carried out with a Loresta GP MCP-T610 resistivity meter (Mitsubishi Chemical Analytech Co., Chigasaki, Kanagawa, Japan) using a MCP-TP06P probe (inter-pin distance 1.5 mm, pin points 0.26 R, spring pressure 70 g pin^−1^). Electrochemical measurements were performed using a BAS CV-50W voltammetric analyzer (Bioanalytical Systems Inc., West Lafayette, IN, USA) and a single-compartment three-electrode cell. A platinum wire and an Ag/AgCl electrode (BAS) served as counter and reference electrodes, respectively. The rod of spectroscopic graphite (SG) electrode with an outer diameter of 3.05 mm (type RW001, Ringsdorff Werke GmbH, Germany) coated with a synthesized polymer/PSS served as a working electrode.

### 3.6. Determination of Minimal Inhibitory Concentration (MIC)

The minimal inhibitory concentration (MIC) of the polymer/PSS complexes was determined by the standard serial two-fold dilution method in LB nutrient medium (Luria-Bertani, Miller, Sigma). The gram-positive bacterium *Staphylococcus aureus* 209P, gram-negative bacterium *E. coli* K 12 and the yeast *Yarrowia lipolytica* 367-2 were used as test microorganisms. 200 µL of earlier prepared sterile media containing various concentrations of polymer/PSS was placed in the wells of a 96-well microplate in 3 replicates for each concentration. Next, each well was inoculated with 4 µL of the test culture during the stationary growth phase (1 day) and incubated at 28 °C with 150 rpm agitation for 24 h. Then the optical density of each well was measured relative to controls without inoculum at 540 nm using an Ao Absorbance Microplate Reader (Azure biosystems, Dublin, CA, USA). The growth of microorganisms was assessed by change in optical density values compared to the initial values (immediately after medium inoculation). MIC was defined as the lowest concentration of a compound that inhibited bacterial growth within 24 h. Each strain was tested in triplicate.

## 4. Conclusions

The present work has demonstrated the possibility of using betain-glycerol DES as a co-solvent for effective laccase catalyzed polymerization/copolymerization of aniline (ANI) and 3-aminobenzoic acid (3ABA) to produce conducting polyanilines. The composition of the reaction medium meets the requirements of sustainable chemistry. The synthesized polymer/PSS complexes are different in morphology and electrochemical activity, and their conductivity decreases in the following way: PANI/PSS > P(ANI-3ABA)/PSS > P3ABA. P(ANI-3ABA)/PSS strongly inhibits the growth of *S. aureus* and *E.coli*. The laccase from the fungus *T. hirsuta* used for polymerization preserves about 50% of activity after incubation for 120 h in the DES/buffer mixture (60/40 vol.%). Thus, betaine-based deep eutectic solvents are promising media for biotransformation of various substrates of laccase, including poorly soluble compounds.

## Figures and Tables

**Table 1:** Characteristics of the redox conversions of the polymer/PSS complexes *.

Polymers	Redox Peak Couple	E_a_, V	E_c_, V	E_mp_, V	ΔE, V
PANI/PSS	I	0.065	–0.129	–0.032	0.194
	II	0.385	0.225	0.305	0.160
P(ANI-3ABA)/PSS	I	0.063	–0.149	–0.043	0.212
	II	0.396	0.258	0.327	0.139
P3ABA/PSS	I	–0.040	–0.260	–0.150	0.220
	II	0.075	0.081	–0.003	0.155

* Cyclic voltammograms were recorded at 50 mV s^−1^.

## Data Availability

Not applicable.

## Supplementary Materials

The following supporting information can be downloaded at: https://www.mdpi.com/article/10.3390/ijms231911409/s1.
