# Supplementary material for: Betaine-Based Deep Eutectic Solvent as a New Media for Laccase-Catalyzed Template-Guided Polymerization/Copolymerization of Aniline and 3-Aminobenzoic Acid
Source: Int J Mol Sci. 2022 Sep 27;23(19):11409. doi: 10.3390/ijms231911409 (PMC9569669; doi:10.3390/ijms231911409)
Supplement: Supplementary file 1 [file ijms-23-11409-s001.zip › ijms-1914003-supplementary.pdf]

Supplementary Materials

Betaine-Based Deep Eutectic Solvent as a New Media for Laccase-Catalyzed Template-Guided Polymerization/Copolymerization of Aniline and 3-Aminobenzoic Acid

IrinaVasil'eva, Olga Morozova, Galina Shumakovich and Alexander Yaropolov *

A. N. Bach Institute of Biochemistry, Research Center of Biotechnology of the Russian Academy of Sciences, Leninsky Ave. 33, bld. 2, 119071 Moscow, Russia

* Correspondence: yaropolov@inbi.ras.ru, alexander-yaropolov52@yandex.ru (A.Y.)

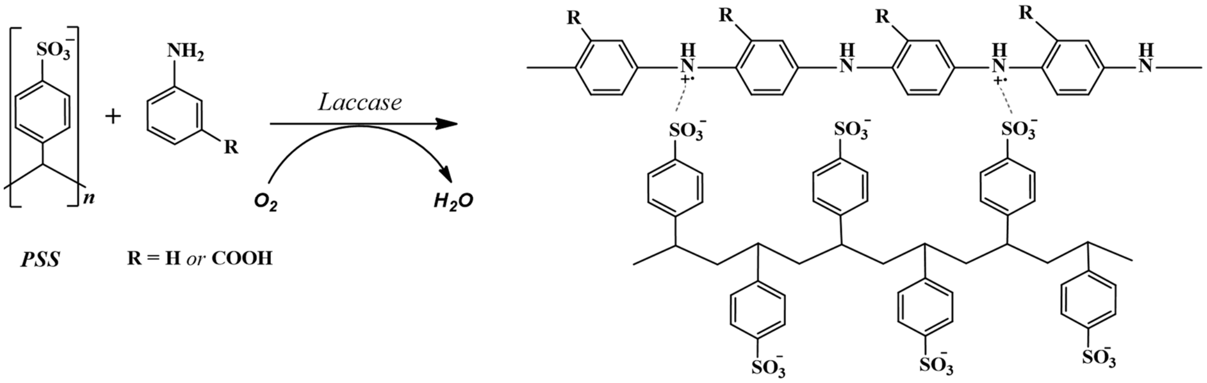

Figure S1. Scheme of the enzymatic polymerization/copolymerization of ANI and 3ABA on a PSS template
